# Supplementary material for: Voxel-based versus network-analysis of changes in brain states in patients with auditory verbal hallucinations using the Eriksen Flanker task
Source: PLoS One. 2025 Mar 20;20(3):e0319925. doi: 10.1371/journal.pone.0319925 (PMC11925307; doi:10.1371/journal.pone.0319925)
Supplement: S1 Table — (DOCX) [file pone.0319925.s001.docx]

**S1 Table**

| **Cluster size** | **Peak t-value** | **Peak z-value** | **X** | **Y** | **Z** | **Anatomical localization** |
| --- | --- | --- | --- | --- | --- | --- |
| 22301 | 11.2 | Inf | -38 | -40 | 38 | Left supramarginal gyrus |
|  | 10.4 | Inf | 30 | -4 | 52 | Right precentral gyrus |
|  | 10.4 | Inf | -6 | 6 | 48 | Left supplementary motor cortex |
| 1814 | 8.5 | 7.3 | 42 | -62 | -18 | Right occipital fusiform gyrus |
|  | 8.3 | 7.2 | 32 | -90 | -6 | Right inferior occipital gyrus |
|  | 8.1 | 7.1 | 30 | -90 | -14 | Right occipital fusiform gyrus |
| 1102 | 8.4 | 7.3 | 40 | 40 | 24 | Right middle frontal gyrus |
| 368 | 7.4 | 6.6 | -2 | -68 | -22 | Cerebellar vermal lobules VI-VII |
|  | 6.6 | 6 | 6 | -66 | -22 | Cerebellar vermal lobules VI-VII |
| 351 | 7.3 | 6.5 | -30 | 18 | 4 | Left anterior insula |
| 351 | 6.3 | 5.8 | 14 | -16 | 4 | Right thalamus Proper |
|  | 4.7 | 4.4 | 8 | -22 | -10 | Right ventral DC |
| 327 | 5.8 | 5.4 | -14 | -20 | -2 | Left thalamus proper |
| 89 | 5.6 | 5.2 | -38 | 32 | 30 | Left middle frontal gyrus |
| 31 | 4.9 | 4.6 | -34 | 44 | 20 | Left middle frontal gyrus |
