# Supplementary material for: Voxel-based versus network-analysis of changes in brain states in patients with auditory verbal hallucinations using the Eriksen Flanker task
Source: PLoS One. 2025 Mar 20;20(3):e0319925. doi: 10.1371/journal.pone.0319925 (PMC11925307; doi:10.1371/journal.pone.0319925)
Supplement: S2 Table — (DOCX) [file pone.0319925.s002.docx]

**S2 Table**

| **Cluster size** | **Peak t-value** | **Peak z-value** | **X** | **Y** | **Z** | **Anatomical localization** |
| --- | --- | --- | --- | --- | --- | --- |
| 52390 | 14.7 | Inf | -6 | 6 | 46 | Left supplementary motor cortex |
|  | 13.6 | Inf | 6 | 6 | 50 | Right supplementary motor cortex |
|  | 13.3 | Inf | -4 | -2 | 52 | Left supplementary motor cortex |
| 433 | 7.1 | 6.3 | -40 | 38 | 30 | Left middle frontal gyrus |
|  | 6.5 | 5.9 | -36 | 44 | 24 | Left middle frontal gyrus |
|  | 6.3 | 5.8 | -34 | 36 | 24 | Left middle frontal gyrus |
