# Supplementary material for: Voxel-based versus network-analysis of changes in brain states in patients with auditory verbal hallucinations using the Eriksen Flanker task
Source: PLoS One. 2025 Mar 20;20(3):e0319925. doi: 10.1371/journal.pone.0319925 (PMC11925307; doi:10.1371/journal.pone.0319925)
Supplement: S3 Table — (DOCX) [file pone.0319925.s003.docx]

**S3 Table**

| **Cluster size** | **Peak t-value** | **Peak z-value** | **X** | **Y** | **Z** | **Anatomical localization** |
| --- | --- | --- | --- | --- | --- | --- |
| 2606 | 5.66 | 5.25 | -12 | -50 | 14 | Left precuneus |
|  | 5.53 | 5.14 | 4 | -88 | 22 | Right cuneus |
|  | 5.53 | 5.14 | 12 | -56 | 10 | Right precuneus |
| 175 | 5.59 | 5.19 | -40 | -80 | 28 | Left middle occipital gyrus |
|  | 3.85 | 3.71 | -50 | -72 | 22 | Left middle occipital gyrus |
|  | 3.52 | 3.41 | -44 | -66 | 20 | Left angular gyrus |
| 15 | 4.03 | 3.86 | 46 | -74 | 28 | Right middle occipital gyrus |
| 10 | 3.46 | 3.35 | 50 | -68 | 20 | Right middle occipital gyrus |
