# Supplementary material for: Voxel-based versus network-analysis of changes in brain states in patients with auditory verbal hallucinations using the Eriksen Flanker task
Source: PLoS One. 2025 Mar 20;20(3):e0319925. doi: 10.1371/journal.pone.0319925 (PMC11925307; doi:10.1371/journal.pone.0319925)
Supplement: S4 Table — (DOCX) [file pone.0319925.s004.docx]

**S4 Table**

| **Cluster size** | **Peak t-value** | **Peak z-value** | **X** | **Y** | **Z** | **Anatomical localization** |
| --- | --- | --- | --- | --- | --- | --- |
| 1771 | 8.4 | 7.3 | -2 | 40 | -6 | Left anterior cingulate gyrus |
|  | 7.9 | 7 | -4 | 54 | -10 | Left medial frontal cortex |
|  | 6.5 | 5.9 | 12 | 46 | -14 | Right cerebral white matter |
| 353 | 8 | 7 | -40 | -80 | 28 | Left middle occipital gyrus |
| 1507 | 6.4 | 5.9 | -8 | -60 | 12 | Left precuneus |
|  | 5.6 | 5.2 | 14 | -56 | 10 | Right precuneus |
| 32 | 4.9 | 4.6 | 48 | -72 | 28 | Right middle occipital gyrus |
| 158 | 4.8 | 4.5 | -8 | -94 | 20 | Left cerebral white matter |
|  | 4.7 | 4.4 | 10 | -90 | 26 | Right cuneus |
|  | 3.8 | 3.6 | 14 | -94 | 16 | Right occipital pole |
| 25 | 4.4 | 4.2 | -24 | -14 | -22 | Left hippocampus |
| 17 | 4.1 | 3.9 | 36 | 12 | -26 | Right temporal pole |
| 231 | 4.1 | 3.9 | -14 | 40 | 52 | Left superior frontal gyrus |
|  | 4.1 | 3.9 | -22 | 32 | 52 | Left superior frontal gyrus |
|  | 3.7 | 3.5 | -22 | 26 | 44 | Left superior frontal gyrus |
| 35 | 4 | 3.8 | 26 | -16 | -24 | Right hippocampus |
| 26 | 3.7 | 3.6 | -8 | 60 | 24 | Left superior frontal gyrus medial segment |
| 39 | 3.6 | 3.5 | -30 | -36 | -20 | Left fusiform gyrus |
|  | 3.6 | 3.4 | -28 | -42 | -14 | Left fusiform gyrus |
