# Supplementary material for: Voxel-based versus network-analysis of changes in brain states in patients with auditory verbal hallucinations using the Eriksen Flanker task
Source: PLoS One. 2025 Mar 20;20(3):e0319925. doi: 10.1371/journal.pone.0319925 (PMC11925307; doi:10.1371/journal.pone.0319925)
Supplement: Attachment — Submitted filename: Response to Reviewers.docx [file pone.0319925.s006.docx]

**Response to reviewer comments, MS. PONE-D-23-36278 by Sandøy et al.**

**Journal Requirements:**

When submitting your revision, we need you to address these additional requirements.

*1. Please ensure that your manuscript meets PLOS ONE's style requirements, including those for file naming. The PLOS ONE style templates can be found at* [*https://journals.plos.org/plosone/s/file?id=wjVg/PLOSOne_formatting_sample_main_body.pdf*](https://journals.plos.org/plosone/s/file?id=wjVg/PLOSOne_formatting_sample_main_body.pdf) *and* [*https://journals.plos.org/plosone/s/file?id=ba62/PLOSOne_formatting_sample_title_authors_affiliations.pdf*](https://journals.plos.org/plosone/s/file?id=ba62/PLOSOne_formatting_sample_title_authors_affiliations.pdf)

**Response:** The formatting of the manuscript has been updated and supporting information has been uploaded as a separate file.

*2. Note from Emily Chenette, Editor in Chief of PLOS ONE, and Iain Hrynaszkiewicz, Director of Open Research Solutions at PLOS: Did you know that depositing data in a repository is associated with up to a 25% citation advantage (*[*https://doi.org/10.1371/journal.pone.0230416*](https://doi.org/10.1371/journal.pone.0230416)*)? If you’ve not already done so, consider depositing your raw data in a repository to ensure your work is read, appreciated and cited by the largest possible audience. You’ll also earn an Accessible Data icon on your published paper if you deposit your data in any participating repository (*[*https://plos.org/open-science/open-data/#accessible-data*](https://c05y1x9s.r.us-east-2.awstrack.me/L0/https:%2F%2Fplos.org%2Fopen-science%2Fopen-data%2F%23accessible-data/1/010f0191de28f99c-e8c88fb5-d4be-4ce7-9dc7-237b66055321-000000/xNSvw9xbm1q3Sg0_tWUPl4y0sFk=176)*).*

**Response:** This is unfortunately not possible for the sensitive data included in the study due to ethical regulations under which the data were collected. Please see the Response under requirement 5., as this addresses the same issue.

*3.* Thank you for stating the following in the Competing Interests section: “*I have read the journal's policy and the authors of this manuscript have the following competing interests: KH, RG, ARC and LE own shares in the NordicNeuroLab Inc,* [*https://www.nordicneurolab.com/*](https://www.nordicneurolab.com/)*, which produced add-on equipment for the fMRI data acquisitions. All authors declare no conflict of interest.”*

*Please confirm that this does not alter your adherence to all PLOS ONE policies on sharing data and materials, by including the following statement: "This does not alter our adherence to  PLOS ONE policies on sharing data and materials.” (as detailed online in our guide for authors* [*http://journals.plos.org/plosone/s/competing-interests*](http://journals.plos.org/plosone/s/competing-interests)*).  If there are restrictions on sharing of data and/or materials, please state these. Please note that we cannot proceed with consideration of your article until this information has been declared.*

*Please include your updated Competing Interests statement in your cover letter; we will change the online submission form on your behalf.*

**Response:** The Competing Interests section should be altered to the following: “We have read the journal's policy and the authors of this manuscript have the following competing interests: KH, RG, ARC and LE own shares in the NordicNeuroLab Inc, https://www.nordicneurolab.com/, which produced add-on equipment for the fMRI data acquisitions. All authors declare no conflict of interest. This does not alter our adherence to PLOS ONE policies on sharing data and materials.”

*4. We noted in your submission details that a portion of your manuscript may have been presented or published elsewhere. [The data for the healthy controls were previously used by our group in the study by Craven et al. (2023, doi:10.1002/nbm.5065).] Please clarify whether this [conference proceeding or publication] was peer-reviewed and formally published. If this work was previously peer-reviewed and published, in the cover letter please provide the reason that this work does not constitute dual publication and should be included in the current manuscript.*

**Response:** While some of our healthy control data were included in previous peer-reviewed work (Craven et al., 2023), that work focused on functional MR spectroscopy (fMRS). Functional imaging data (fMRI) was only used for technical validation of the fMRS findings. It therefore does not constitute a dual publication.

The following statement was added to the manuscript on page 6 under the section Participants: *“ …some of the data for the healthy controls were included in a previous publication by Craven et al. [32] for technical validation of functional MR spectroscopy findings.”*

*5. We note that you have indicated that there are restrictions to data sharing for this study. PLOS only allows data to be available upon request if there are legal or ethical restrictions on sharing data publicly. For more information on unacceptable data access restrictions, please see* [*http://journals.plos.org/plosone/s/data-availability#loc-unacceptable-data-access-restrictions*](http://journals.plos.org/plosone/s/data-availability#loc-unacceptable-data-access-restrictions)*.*

*Before we proceed with your manuscript, please address the following prompts:*

*a) If there are ethical or legal restrictions on sharing a de-identified data set, please explain them in detail (e.g., data contain potentially identifying or sensitive patient information, data are owned by a third-party organization, etc.) and who has imposed them (e.g., a Research Ethics Committee or Institutional Review Board, etc.). Please also provide contact information for a data access committee, ethics committee, or other institutional body to which data requests may be sent.*

*b) If there are no restrictions, please upload the minimal anonymized data set necessary to replicate your study findings to a stable, public repository and provide us with the relevant URLs, DOIs, or accession numbers. For a list of recommended repositories, please see*

[*https://journals.plos.org/plosone/s/recommended-repositories*](https://journals.plos.org/plosone/s/recommended-repositories)*. You also have the option of uploading the data as Supporting Information files, but we would recommend depositing data directly to a data repository if possible.*

*We will update your Data Availability statement on your behalf to reflect the information you provide. If there are no restrictions, please upload the minimal anonymized data set necessary to replicate your study findings to a stable, public repository and provide us with the relevant URLs, DOIs, or accession numbers.*

**Response:** This is addressed in the Data Availability statement, we have updated the statement to address the ethical restrictions and data sharing restrictions.

*“The data analyzed in this study is subject to the following licenses/restrictions: Due to restrictions posed by the ethics approval (REK Vest #2016/800), data are not allowed shared other than after request to the corresponding author, and after written permission from the Regional Committee for Medical Research Ethics in Western Norway (REK Vest). Requests to access these datasets should be directed to LBS,* [*lydia.sandoy@uib.no*](mailto:lydia.sandoy@uib.no)*.”*

*6. We notice that your supplementary [S1-S4 Table] are included in the manuscript file. Please remove them and upload them with the file type 'Supporting Information'. Please ensure that each Supporting Information file has a legend listed in the manuscript after the references list.*

**Response:** The Tables are now in a separate file named “Supporting Information”, and the table legends are included in the manuscript after the reference list.

**Comments to the Author**

1. Is the manuscript technically sound, and do the data support the conclusions?

The manuscript must describe a technically sound piece of scientific research with data that supports the conclusions. Experiments must have been conducted rigorously, with appropriate controls, replication, and sample sizes. The conclusions must be drawn appropriately based on the data presented.

Reviewer #1: No

Reviewer #2: Yes

2. Has the statistical analysis been performed appropriately and rigorously?

Reviewer #1: Yes

Reviewer #2: Yes

3. Have the authors made all data underlying the findings in their manuscript fully available?

The [PLOS Data policy](http://c05y1x9s.r.us-east-2.awstrack.me/L0/http:%2F%2Fwww.plosone.org%2Fstatic%2Fpolicies.action%23sharing/1/010f0191de28f99c-e8c88fb5-d4be-4ce7-9dc7-237b66055321-000000/z3Zf1myLLit7TNz1Rvo70T03tKg=176) requires authors to make all data underlying the findings described in their manuscript fully available without restriction, with rare exception (please refer to the Data Availability Statement in the manuscript PDF file). The data should be provided as part of the manuscript or its supporting information, or deposited to a public repository. For example, in addition to summary statistics, the data points behind means, medians and variance measures should be available. If there are restrictions on publicly sharing data—e.g. participant privacy or use of data from a third party—those must be specified.

Reviewer #1: No

Reviewer #2: No

4. Is the manuscript presented in an intelligible fashion and written in standard English?

PLOS ONE does not copyedit accepted manuscripts, so the language in submitted articles must be clear, correct, and unambiguous. Any typographical or grammatical errors should be corrected at revision, so please note any specific errors here.

Reviewer #1: Yes

Reviewer #2: Yes

**Reviewers’ comments to the author**

**Reviewer #1:**

*1. The authors compared 50 patients with auditory hallucinations and 54 matched healthy controls using fMRI during performance of a cognitive task (the flanker task), which requires ignoring of irrelevant information.*

***Response:*** The participants were 54 patients with mental disorders experiencing mild to severe auditory verbal hallucinations, and 54 sex and age-matched healthy controls. Out of the initially scanned participants, 4 patients were excluded due to incomplete scanning sessions. Thus, **t**he authors compared 50 patients with AVHs and 50 matched healthy controls.

*2. The patient group was heterogenous: while 32 had schizophrenia/schizoaffective disorder, the remainder had a variety of other diagnoses (organic hallucinosis, drug-induced psychosis, acute paranoid psychosis, unspecified non-organic psychosis, bipolar disorder, depressive disorder, personality and no or unknown diagnosis). Comparisons of task-related activations were carried out and also a functional connectivity analysis.

This study has two serious flaws. The first is the marked heterogeneity of the sample in terms of diagnosis. This represents an unusual strategy for studies in this field and severely limits the conclusions that can be drawn; inclusion of patients with organic psychosis, drug induced psychosis and unknown or no diagnoses are of particular concern here. The second is that comparing individuals with schizophrenia with auditory hallucinations and healthy controls is an inappropriate comparison - changes found could well be due to having schizophrenia rather than being related to presence of a particular symptom (in this case hallucinations). The appropriate comparison is between patients with a disorder who have/do not have a particular symptom. Clearly, this issue is complicated by the fact that the diagnosis was not schizophrenia in all the patients the authors included, but the underlying principle is the same.*

**Response:** This is discussed in detail in the Discussion on page 20. We point out in the manuscript that the patient group is heterogeneous with regard to diagnoses. We also address that the literature on schizophrenia patients describes the group as very diverse and heterogeneous. Thus, we have chosen to focus on an ICD-10 symptom that the patient group (mostly F20) has in common across the different sub-diagnoses, rather than a single diagnosis. One could argue that it could be easier to isolate AVHs in a diverse sample because other effects are canceled out. Clinical misclassifications are always an issue with these complex diagnoses, therefore focusing on a symptom that is common across diagnoses is not in our view unreasonable.

The added section is: *“The patient group is diverse and heterogeneous regarding diagnoses, and clinical misclassifications are always an issue with these complex diagnoses. One could argue that it could be easier to isolate AVH in a diverse sample because other effects are canceled out. Thus, we chose to focus on a symptom that the patient group (mostly F20) has in common across the different sub-diagnoses, rather than a single diagnosis.”*

We have restructured the Introduction to focus on AVH to make it more clear that the manuscript is about the symptom AVHs. Then we report the prevalence of the symptom in various diagnoses and schizophrenia.

**Reviewer #2:**

*In their manuscript „Voxel-based versus network-analysis of changes in brain states in patients with auditory verbal hallucinations using the Eriksen Flanker task“, Lydia Brunvoll Sandøy and co-authors set out to investigate neural correlates of switching between task-processing and periods of rest in a block-design in patients with auditory verbal hallucinations (AVHs) and healthy controls. They test cognitive flexibility and conflict control by use of a version of the Eriksen Flanker task in an fMRI block design alternating task engagement and rest.
Results show significant behavioural effects between patients and controls, with patients performing less accurately and with longer reaction times (RTs) than healthy controls. While both groups were shown to recruited similar networks during task and rest, controls displayed increased network variability across task-present and task-absent conditions, indicating that controls were better at switching between networks and conditions, thus explaining their better performance.

The topic of the study is timely and should be of interest to the broad readership of PLOS ONE. Furthermore, it has clinical relevance since, as the authors correctly state, the underlying mechanisms of AVH are still poorly understood.

Introduction
1. In general, the introduction is well written and provides a good overview over the topic. However, the description of the DMN, the EMN and their interaction in healthy participants should be described in more detail to give appropriate background. Is there general agreement on how DMN and EMN interact with each other during switching from task to rest periods? Are there any previous findings on DMN / EMN interaction in patients suffering from AVH?*

**Response:** We have added more about this in the Introduction on page 4:

” *The default mode network (DMN) is a collection of different brain regions that are commonly upregulated during passive rest with intrinsically driven modes of cognition [20]. This is in contrast to the extrinsic mode network (EMN,) which is a generalized task-positive network that involves brain regions that are often shown to be upregulated during diverse cognitive tasks [21]. This dynamic up- and down-regulation of the DMN and EMN has previously been reported for both auditory [22], and visual tasks[23]. Patients with schizophrenia have been shown to have lower activation of a task-positive network, and increased activation in areas associated to the DMN [24, 25]. Similarly, a study by Nygård et al. [26] reported that patients with schizophrenia failed to up-regulate task-positive networks and down-regulate the DMN during an auditory dichotic listening task. However, little is known about the interaction of these neural networks in patients experiencing AVHs.”*

*2. Also, I’m missing a motivation as to why the Eriksen Flanker task was used here (as opposed to a variety of other tasks that have been used to study AVHs.*

**Response:** We have added more information about why the Flanker task was used in our study in the Method section on page 5. The following was added: *“The Flanker task is a classic task to investigate higher cognitive functions, with a large literature of its own. It has also been studied in schizophrenia and psychosis where it has been shown that patients with schizophrenia are impaired compared to healthy controls when solving the task. Using a visual task allowed us to go beyond the auditory modality inherent in AVH experiences, to capture higher cognitive functioning.”*

*3. Given the title of the present manuscript the introduction should also provide some motivation as to why both voxel-based and network-analysis are employed here and advantages / disadvantages of both approaches should be eluted to.*

**Response:** We have added the following to the Introduction on page 5: *“The voxel-based approach and network-analysis was used, as the former does not address interactions and network connectivity, while the network-analysis may have difficulty in examining the contribution of different nodes. An analogy for this is that the voxel-based approach allows us to identify individual steps, whereas the network-analysis reveals how these steps come together to form the overall structure of the staircase. By integrating these approaches, we can gain a comprehensive understanding of the dataset, effectively addressing the limitations inherent in each method.*

*4. Importantly, clear hypotheses based on the literature are missing at the end of introduction.*

**Response:** The following hypotheses were added to the end of the Introduction on page 6:

*“We hypothesized that patients behaviorally would perform the Flanker task slower (RT) compared to controls and that task accuracy is similar to healthy controls. We assume that AVH is a failure of top-down control of bottom-up perceptual processes which could be due to aberrant up- and down regulation of brain networks. Thus, we hypothesized that patients and controls differ significantly in active brain regions and use different networks during task- and rest periods.”*

*5. Minor points: What did Pappa et al. find when comparing patients with schizophrenia to patients with Parkinson’s disease and healthy controls?*

Response: This have now been addressed in the Introduction on page 4:

*“The study by Pappa et al. [30] compared patients with schizophrenia to patients with Parkinson’s disease, where the former had reduced activation of the prefrontal and cingulate cortex indicating prefrontal hypofunction in patients with schizophrenia.”*

*Methods
6. Top of page 7: “All participants were included in the fMRI region-analysis, one patient and the corresponding control were excluded from the network-analysis due to an incomplete structural MR scan, and one patient were excluded due to no recorded response for the behavioural analysis.”
- Please correct typo: were / was
- If two patients were excluded, why are there still 50 patients in the voxel-based analysis group?*

**Response:** We apologize that this was unclear in the manuscript. We have now included a better description on page 8: *“All participants were included in the voxel-based analysis (N=50), one patient and the corresponding control were excluded from the network-analysis due to an incomplete structural MR scan (N=49), and one patient was excluded due to no recorded responses for the behavioral analysis (N=49).”*

7. - Most importantly: Do the results look the same when the same 49 patients are included in the voxel-based and network analysis?

**Response:** Yes, the results are the same when 49 patients and matched controls are included in the voxel-based and network-based analysis. The clusters are the same, although the number of voxels in each cluster from the voxel-based analysis changed minimally with 49 subjects instead of 50.

*8. Minor points: - “Only patients with a score of 3 or higher on the positive subscale score item 3 (P3 hallucinatory behavior) in the PANSS were recruited to the study.”: Did all these patients actually experience AUDITORY hallucinations?*

**Response:** The subscale P3 is related to hallucinatory behavior, but with a clear focus on auditory hallucinations. We are certain that all participants had auditory hallucinations but cannot be sure that all had auditory verbal hallucinations. In the interview, the clinical nurse only recruited patients if they had auditory hallucinations.

This has been added to the methods section on page 6: *“The project nurse recruited patients who experienced auditory hallucinations.”*

*9.- Why were different TRs used for the Task and RS session?*

**Response:** This must have been unclear in the manuscript and has been corrected. During the fMRI-BOLD block session, rest- and task-blocks were interleaved. These blocks were used for the voxel-based and the connectivity-based analyses. Therefore, the TR was the same for task- and RS-blocks. The pure resting-state fMRI session that was included in the data collection was not analyzed in the current study and consequently not reported in the manuscript.

This has been added to the Methods section on page 9: *“This session was not used for analysis in the current paper.”*

*Results:
10. In the behavioural analysis, a sex difference in performance is identified, indicating that female patients’ performance is particularly low. It would be highly interesting to see whether this difference in performance is reflected in brain activation and connectivity patterns as well.*

**Response:** We investigated this further and conducted two-sample t-tests for the voxel-based analysis and the network-based analysis.

For the voxel-based analysis the first t-test compared female patients to female controls for task exclusive activation (Task - Rest), and there were no significant differences between the groups. The second t-test compared female patients to male patients during task exclusive activation, using uncorrected results with a p-value threshold of 0.001 six small clusters was significantly different in the male patient group. The clusters were in the Left lingual gyrus (14 voxels), Right supramarginal gyrus (48 voxels), Right middle temporal gyrus (32 voxels), Right occipital fusiform gyrus (16 voxels), Left cerebellum exterior (40 voxels) and Cerebellar Vermal Lobules VI-VII (56 voxels).

The first two-sample t-test for the network-analysis compared female patients to female controls during the task condition. This resulted in significant differences between the groups with small t-values (±3.75). Female patients had significant correlations between nodes of the dorsal attention network and the fronto-parietal network, while controls had significant correlations between the DMN and the fronto-parietal network. The second t-test compared female patients to male patients during task blocks, finding no significant differences between the groups. We consider that these findings are minor and inconclusive and have therefore not included them to maintain the readability of the manuscript as is.  *9. Minor points:- For reader who are not familiar with the depiction of connectivity patterns as connectivity rings it might be helpful to describe in more detail what exactly these figures show.*

**Response:** We agree and the following description of the connectivity patterns has been added to the Results section on page 15:
*“Each color line in the wheel represents significant t-values between the corresponding nodes, based on the average connectivity above (red) or below (blue) a given zero-level.”*

*Discussion:
10. Behavioural findings in the present study contradict previous findings from a meta-analytic study (Westerhausen et al.). It seems unlikely that this is only due to inclusion criteria. Could the authors please comment on further factors that might explain this discrepancy?*

**Response:** This is now discussed further in the Discussion on page 18:

*“Another reason for the discrepancy in our study, could be due to differences in the analyses performed. The meta-analysis by Westerhausen et al. [28] calculated the effect size of mean differences between patients and controls for incompatibility slowing, while the analysis in the current paper focused on accuracy scores and RT related to accuracy.”*

*11. Altogether the discussion appears somewhat superficial. In particular, the voxel-based and network analyses should be discussed in conjunction rather than separately. Given the mostly unexpected brain imaging findings, further studies should be suggested that might shed more light on how neural correlates of cognitive control differ between patients and controls. Finally, a discussion of the limitations of the present study (which can also explain the null findings) is completely missing.*

**Response:** The Discussion has been restructured. A section about limitations and future studies has been added on page 20:

*“The main limitation of our study is the heterogeneity of the patient group. However, diversity and heterogeneity regarding diagnoses and possibilities for clinical misclassifications are always an issue with these complex diagnoses. On the other hand, one could argue that it would be easier to isolate AVHs in a diverse sample because other effects are canceled out. Thus, we chose to focus on a symptom that the patient group (mostly ICD-10 F20) has in common across the different sub-diagnoses, rather than a single diagnosis.*

*In our study, we used two different analyses to investigate how neural correlates of cognitive control during the Flanker task differ in patients with AVHs. However, future studies should investigate the dynamic up- and down regulation of networks between task and rest blocks, since such an approach could clarify whether patients show hyper- or hypo-activation of networks during changing environmental demands. Future studies could also explore additional visual tasks, to investigate if executive functions in the visual domain are globally affected in patients with AVHs, having the same patients conduct different tasks during fMRI, thus making it possible to compare task performance across tasks.”*

Altogether, this is a relevant study on a topic well suited for publication in PLOS ONE. Therefore, I would recommend publication of the manuscript, if my concerns can be adequately addressed by the authors.

6. PLOS authors have the option to publish the peer review history of their article ([what does this mean?](https://c05y1x9s.r.us-east-2.awstrack.me/L0/https:%2F%2Fjournals.plos.org%2Fplosone%2Fs%2Feditorial-and-peer-review-process%23loc-peer-review-history/1/010f0191de28f99c-e8c88fb5-d4be-4ce7-9dc7-237b66055321-000000/AfJsRh2hScFbLRUe1jRvLRZXhpU=176)). If published, this will include your full peer review and any attached files.

If you choose “no”, your identity will remain anonymous but your review may still be made public.

**Do you want your identity to be public for this peer review?** For information about this choice, including consent withdrawal, please see our [Privacy Policy](https://c05y1x9s.r.us-east-2.awstrack.me/L0/https:%2F%2Fwww.plos.org%2Fprivacy-policy/1/010f0191de28f99c-e8c88fb5-d4be-4ce7-9dc7-237b66055321-000000/DLASsL1BSjjzxD6yaG0O1iA6GLw=176).

Reviewer #1: No

Reviewer #2: No

[NOTE: If reviewer comments were submitted as an attachment file, they will be attached to this email and accessible via the submission site. Please log into your account, locate the manuscript record, and check for the action link "View Attachments". If this link does not appear, there are no attachment files.]

While revising your submission, please upload your figure files to the Preflight Analysis and Conversion Engine (PACE) digital diagnostic tool, [https://pacev2.apexcovantage.com/](https://c05y1x9s.r.us-east-2.awstrack.me/L0/https:%2F%2Fpacev2.apexcovantage.com%2F/1/010f0191de28f99c-e8c88fb5-d4be-4ce7-9dc7-237b66055321-000000/eGFV009km4f_oZlgtdYNFGHMHfU=176). PACE helps ensure that figures meet PLOS requirements. To use PACE, you must first register as a user. Registration is free. Then, login and navigate to the UPLOAD tab, where you will find detailed instructions on how to use the tool. If you encounter any issues or have any questions when using PACE, please email PLOS at [figures@plos.org](mailto:figures@plos.org). Please note that Supporting Information files do not need this step.
